# Clinical observations on intensive immunosuppressive therapy combined with umbilical cord blood support for the treatment of severe aplastic anemia

**DOI:** 10.1186/1756-8722-4-27

**Published:** 2011-06-10

**Authors:** Fang Zhou, Linfu Ge, Zhe Yu, Yuan Fang, Fansheng Kong

**Affiliations:** 1 Department of Hematology, General Hospital of Jinan Military Area, Jinan, Shandong, China

## Abstract

**Objective:**

To evaluate the efficacy of enhanced, intensive, immuno-suppressive therapy with umbilical cord blood support for severe aplastic anemia (SAA).

**Methods:**

A total of 25 patients with SAA received enhanced, intensive, immuno-suppressive therapy and a cord blood transfusion. Therapy protocol: Anti-thymocyte globulin (ATG) 2.5 mg/(kg•d) × 5d; Cyclophosphamide 50 mg/(kg•d) × 2d; cyclosporin A (CsA) maintenance therapy.

**Result:**

25 patients were enrolled. 18 underwent a complete recovery, 4 made significant improvements, 1 did not respond, and 2 died. Therefore, the efficacy rate was 88%. The median follow-up time was 35 months (range 13-47 months), and the 3-year overall survival rate was 92%. Patients rapidly achieved reconstitution of hematopoiesis. The median time to neutrophil ANC > 0.5 × 10^9^/L was 18 days (range 8-36), platelets >20 × 10^9^/L was 34 days (range 12-123), and Hb > 100 g/L 95 dyas (range 35-173).

**Conclusion:**

Enhanced, intensive, immuno-suppressive therapy with umbilical cord blood support may be an effective option for SAA therapy.

## 

To the Editor:

Severe aplastic anemia (SAA) has a high mortality rate[1]. High-dose cyclophosphamide (CTX) treatment for SAA provides some benefit; however, the recovery of hematopoiesis is slow, and some studies have demonstrated high treatment-related mortality rates. Therefore, the toxic side effects of high-dose CTX (e.g., hemorrhagic cystitis) cannot be ignored. Intensive immunosuppressive therapy, such as combined anti-thymocyte globulin (ATG) and cyclosporine (CSA), has an average onset of efficacy of 3-7 months and an efficacy rate of 60-80%. Before the onset of efficacy, patients are susceptible to severe infection. Moreover, infection shortly after treatment is a major cause of death in these patients and requires large number of blood transfusions[2,3]. In 1974, Knudtzon et al[4]. first discovered hematopoietic progenitor cells in umbilical cord blood. After cord blood transfusion, early hematopoietic progenitor cells from umbilical cord blood can survive, proliferate, and differentiate in a patient's body for a short time. During this time, they can secrete hematopoietic stimulating factors and contribute to hematopoietic replacement, which both shortens the duration of and helps patients to overcome agranulocytosis[5]. There is a reduced requirement for transfusion of blood products and an increase in the total efficacy rate for this treatment.

In this study, 25 patients with severe aplastic anemia were treated with intensive immunosuppressive therapy combined with supportive therapy of umbilical cord blood transfusion. All patients were treated in our department between January 2006 and January 2009. Patients were confirmed to have SAA by hemogram analysis and bone marrow biopsies. In total, 25 patients between the ages of 3 and 28 were included (median age: 11 years old). Of these patients, 12 were male and 13 were female, and there were 12 patients >12 yrs, and 13 <= 12 yrs. The patients were treated with intensive immunosuppressive agents combined with an umbilical cord blood transfusion.

## Treatment regimen

ATG 2.5-3.0 mg/(kg·d) × 5d and cyclophosphamide CTX 50 mg/(kg·d) × 2d + cyclosporine A were combined with an unrelated umbilical cord blood transfusion. The mononuclear cell (MNC) count of the transfused cord blood was 2.25-15.1 × 10^7 ^cells/kg with a median MNC count of 4.0 × 10^7 ^cells/kg. Two patients weighing over 80 kg were given double units of the umbilical cord blood transfusion. HLA matching and blood typing were performed for umbilical cord blood selection, and cord blood units with 1-3 HLA typing mismatches were selected. G-CSF (5 μg/kg·d) was given until the absolute neutrophil count (ANC) was > 1.5 × 10^9 ^cells/L. To enhance platelets recovery, all patients received 1.5 mg/day of IL-11.

## Results

Of the 25 patients, 18 underwent a complete recovery, 4 made significant improvements, 1 did not respond, and 2 died. Therefore, the efficacy rate was 88%. The median follow-up time was 35 months (range 13-47 months), and the 3-year overall survival rate was 92%.

Patients rapidly achieved reconstitution of hematopoiesis. The median time to neutrophil ANC > 0.5 × 10^9^/L was 18 days (range 8-36), platelets >20 × 10^9^/L was 34 days (range 12-123), and Hb > 100 g/L 95 days (range 35-173). Although the body weights between age groups (< = 12 yrs vs >12 ) differ significantly, the hematopoiesis recovery time did not significantly differ between the two groups (Table 1) (*P *> 0.05 using a *t*-test). There was no durable donor engraftment nor GVHD. Therefore the umbilical cord blood transfusion provided transient hematopoietic support and reduced transfusion requirement. Further expanded study is needed to characterize the kinetics of hematopoietic reconstitution.

**Table 1:** Recovery of hematopoiesis in different age groups

			hematopoiesis reconstitution (range days)		
			
Age Group	Number of cases	**Median MNC 10**^**7 **^**/kg**	**ANC > 0.5 × 10**^**9**^**/L**	**PLT > 20 × 10**^**9**^**/L**	Hb > 100 g/L
**≤12 yrs**	13		19 (9-34)	45 (12-74)	89 (34-156)
**>12 yrs**	12	4.0	22 (8-38)	53 (15-123)	107 (35-173)

## List of Abbreviations

SAA: Severe Aplastic Anemia; CsA: Cyclosporin A; ANC: Absolute Neutrophil Count; ATG: Anti-thymocyte Globulin; CTX: Cyclophosphamide; G-CSF: Granulocyte Colony-Stimulating Factor; MNC: Mononuclear cell; GVHD: Graft Versus Host Disease

## Conflict of interests

The authors declare that they have no competing interests.

## Authors' contributions

FZ performed the clinical observations procedures, designed and coordinated the study, interpreted data and wrote the manuscript; LG collected patient data and samples, assisted with statistical analysis and data interpretation; ZY collected patient data and samples; YF collected patient data and samples; FKperformed the statistical analysis.

All authors have read and approved the final manuscript.
